# High Efficient Visible-Light Photocatalytic Performance of Cu/ZnO/rGO Nanocomposite for Decomposing of Aqueous Ammonia and Treatment of Domestic Wastewater

**DOI:** 10.3389/fchem.2018.00219

**Published:** 2018-06-12

**Authors:** Shiying He, Pengfu Hou, Evangelos Petropoulos, Yanfang Feng, Yingliang Yu, Lihong Xue, Linzhang Yang

**Affiliations:** ^1^Institute of Agricultural Resources and Environment, Jiangsu Academy of Agricultural Sciences, Nanjing, China; ^2^School of Engineering, Newcastle University, Newcastle upon Tyne, United Kingdom

**Keywords:** ZnO, graphene oxide, nanocomposite, photocatalysis, ammonia-nitrogen removal, water treatment

## Abstract

Photocatalytic removal of ammonium-nitrogen (${\text{NH}}_{4}^{+}$-N) from water using solar energy is an approach of high interest and applicability due to the convenience in application. ZnO has a great potential in photocatalytic decomposition of ${\text{NH}}_{4}^{+}$-N and conversion of this nutrient to under visible light irradiations. However the applicability of pristine ZnO though is limited due to its reduced capacity to utilize light from natural light. Herein, we report a two-step ZnO-modified strategy (Cu-doped ZnO nanoparticles, immobilized on reduced graphene oxide (rGO) sheets) for the promotion of photocatalytic degradation of ${\text{NH}}_{4}^{+}$-N under visible light. UV-Vis spectra showed that the Cu/ZnO/rGO can be highly efficient in the utilization of photons from the visible region. Hence, Cu/ZnO/rGO managed to demonstrate adequate photocatalytic activity and effective ${\text{NH}}_{4}^{+}$-N removal from water under visible light compared to single ZnO. Specifically, up to 83.1% of ${\text{NH}}_{4}^{+}$-N (initial concentration 50 mg·L^−1^, catalyst dosage 2 g·L^−1^, pH 10) was removed within 2 h retention time under Xe lamp irradiation. From the catalysis, the major by-product was N_2_. The high ammonia degradation efficiency from the ZnO/Cu/rGO is attributed to the improvement of the reactive oxygen species (ROSs) production efficiency and the further activation of the interfacial catalytic sites. This study also demonstrated that such nanocomposite is a recyclable agent. Its ${\text{NH}}_{4}^{+}$-N removal capacity remained effective even after five batch cycles. In addition, Cu/ZnO/rGO was applied to treat real domestic wastewater, and it was found that chemical oxygen demand (COD), total nitrogen (TN) and total phosphorus (TP) removal efficiencies can reach 84.3, 80.7, and 90.3%, respectively. Thus, Cu/ZnO/rGO in the presence of solar light can be a promising photocatalyst in the field of wastewater treatment.

## Introduction

Accumulation of ammonia nitrogen (${\text{NH}}_{4}^{+}$-N) to aqueous ecosystems, as a result of the rapid agricultural and industrial development (Kirkpatrick et al., 2014; Li et al., 2014), severely impacts the aquatic ecosystems due to eutrophication. Specifically, increased presence of ammoniacal compounds in water leads to cyanobacteria blooms harmful to various aquatic species due to their competitive behavior for respiration (Hued et al., 2006). Furthermore, the ammoniacal oxidative forms (${\text{NO}}_{2}^{-}$, ${\text{NO}}_{3}^{-}$) are harmful to human health (Sun et al., 2012). Numerous techniques have been developed to treat ammonia-rich wastewater including biological processing (Li et al., 2016), constructed wetlands (Wang et al., 2016), adsorption (Halim et al., 2010), chemical oxidation (Kurniawan et al., 2006), and photocatalytic processes (Meeroff et al., 2012). Among these, photocatalytic removal of aqueous ${\text{NH}}_{4}^{+}$-N has been extensively investigated due to its high efficiency, low cost, and the absence of secondary pollutants (Nemoto et al., 2007; Yuzawa et al., 2012).

Semiconductor photocatalysts such as TiO_2_, ZnO, g-C_3_N_4_, and Bi_2_O_3_ have attracted enormous research interest in environmental pollution in contiguous years (Hao et al., 2017; Ma et al., 2018; Zhang et al., 2018). Among various semiconductor photocatalysts, ZnO is considered as one of the most promising photocatalysts for solar energy conversion and photodegradation of pollutants. Its advantages are the high oxidative capacity, low toxicity, high abundance at low cost, and optical stabilities (Daneshvar et al., 2007b; Kuriakose et al., 2015; Akir et al., 2017). When ZnO is illuminated with UV light an electron (e^−^) excites from the valence band (VB) to the conduction band (CB) leaving a hole (h^+^) in the VB, generating electron-hole pairs (Equation 1) (Lee et al., 2016). Holes are powerful oxidants that can be excited to split water molecules producing hydroxyl radicals (•OH) (Equation 2), whereas electrons can act as O_2_ reductants for hydroxyl radicals (•OH) formation (Equations 3, 4) (Lee et al., 2016). Both •OH and holes could oxidize ammonia to nitrogen gas (Reaction Scheme I) and/or to nitrate (Reaction Scheme II) (Altomare et al., 2012). Reaction Scheme I is the ideal path for ammonia conversion due to the production of harmless N_2_ and H_2_ gases. The production of H_2_ also highlights the capacity of this photocatalytic process for carbon neutral/positive “waste” water treatment and/or bio-refinement.

(1) $$\begin{array}{l} ZnO+hv\to ZnO\left({e_{cb}}^{-}+{h_{vb}}^{+}\right) \end{array}$$

(2) $$\begin{array}{l} h^{+}+H_{2}O\to H^{+}+\cdot OH \end{array}$$

(3) $$\begin{array}{l} O_{2}+e^{-}+H^{+}\to H_{2}O_{2} \end{array}$$

(4) $$\begin{array}{l} H_{2}O_{2}+e^{-}\to \cdot OH+OH^{-} \end{array}$$

Reaction Scheme I:

(5) $$\begin{array}{l} NH_{4}+\cdot OH\to {\cdot NH}_{2}+H_{2}O \end{array}$$

(6) $$\begin{array}{l} {\cdot NH}_{2}+\cdot OH\to NH+H_{2}O\left(or\;H^{+}\right) \end{array}$$

(7) $$\begin{array}{l} NH+\cdot OH\to N+H_{2}O\left(or\;H^{+}\right) \end{array}$$

(8) $$\begin{array}{l} NH_{x}+NH_{y}\to N_{2}H_{x+y}\left(x,y=\;0,1,2\right) \end{array}$$

(9) $$\begin{array}{l} {N_{2}H}_{x+y}+\left(x+y\right)h^{+}\to N_{2}+\left(x+y\right)H^{2}\left(N_{2}\;formation\right) \end{array}$$

Reaction Scheme II:

(10) $$\begin{array}{l} NH_{3}+OH^{\cdot }\to NH_{2}OH+H^{+} \end{array}$$

(11) $$\begin{array}{l} NH_{2}OH+OH^{\cdot }\to NO_{2}^{-}\to NO_{3}^{-} \end{array}$$

The applicability of pristine ZnO for photocatalysis purposes is limited due to the reduced light utilization from natural light (~4% of the solar spectrum) as a result of its wide band gap (3.37 eV). Moreover, the photocatalytic efficiency of ZnO is often hindered by the fast charge recombination of the photoinduced electron-hole pairs (h^+^/e^−^) and the lack of active sites on the catalyst surface (Daneshvar et al., 2007a; Akir et al., 2017). Thus, development of efficient procedures to overcome these bottlenecks and promote the light absorption from the visible-light region is essential. Numerous studies have tried to develop a low-cost, highly-active, and easily recycled photocatalysts. Various approaches were employed to achieve that including doping of cations and anions, creating oxygen vacancies, semiconductor coupling, and combining with carbon materials (Hao et al., 2016; Lee et al., 2016; Qi et al., 2017; Lu et al., 2018). Additions of Cu^2+^ have been reported as a promising method to decrease the band gap (Kuriakose et al., 2015; Yildirim et al., 2016). Copper ions have the similar ionic radii (0.73 Å) to the ionic radii of Zn (0.74 Å), which can easily form extra impurities at the ZnO lattice and add a localized electronic energy level within the band gap. The dopant energy level is temporally located below the CB, which subsequently facilitates electron trapping and inhibits the recombination of the h^+^/e^−^ pairs. It is observed that the photocatalytic efficiency and the absorption in visible-light enhances with Cu^2+^ doping (Sharma et al., 2017).

Regardless the potential benefits of the Cu doped ZnO, its efficiency as a visible-light photocatalyst is yet limited. Reports have shown that addition of carbon materials (such as carbon nanotubes, graphene, amorphous carbon) could improve the stability and photocatalytic performance of ZnO, not only by enhancing charge separation, but also by extending its optical absorption to the visible light range. Graphene oxide (GO) [two-dimensional (2D) carbon nanostructure] is a promising additive as it possesses high electron mobility (exceeding 2,000 cm^2^/V), good optical transparency, high surface area (2,600 m^2^ g^−1^) (Bolotin et al., 2008; Jang et al., 2013) and contains series of reactive oxygen functional groups on their surface (e.g., carboxylic acid, hydroxyl, and epoxide groups) (Akir et al., 2017). All these properties make GO one of the most desirable materials for photocatalyst support (Sun et al., 2015). ZnO/GO composite has been previously fabricated using hydrothermal and electrochemical methods with improved photodegradation performance under visible light irradiation. The 2D GO acts both as an electron-acceptor as well as an electron-transport material that manipulates the charge transfer across the ZnO-graphene interface. The electrons transfer from the ZnO conduction band to the GO energy level can reside there for longer periods (Zhao et al., 2017). This phenomenon results in effective separation of the photo-generated carriers that often inhibit the h^+^/e^−^ recombination and in their absence the photocatalytic activity of ZnO can be drastically enhanced (Hsieh and Ting, 2018).

Many studies demonstrated improved ZnO photocatalytic performance by either doping or via GO addition for the enhancement of light absorption and recombination reduction. Therefore, combining the advantage of Cu doping with those of graphene support, Cu/ZnO/rGO nanocomposite could in theory promote visible-light-driven photocatalysis. Thus far, our knowledge on applications of Cu/ZnO/rGO composite for catalysis is limited, especially in the case of nutrient treatment from water. To shed further light on this field we fabricated a Cu/ZnO/rGO nanocomposite to investigate the photocatalytic activity of this specific nanocomposite to ammonia decomposition. Its practical application in the treatment of real domestic wastewater was also evaluated.

## Materials and methods

### Synthesis of Cu/ZnO/rGO photocatalyst

Cu/ZnO/rGO nanocomposite was prepared via a two-step hydrothermal method. Zinc acetate hydrate (50 mL, 0.34 M), copper nitrate hydrate (50 mL, 0.015 M) and sodium oxalate (40 mL, 0.5 M) were mixed (magnetic stirrer) at room temperature until homogeneity was achieved. KOH (20 mL, 1 M) was then added drop wise. After stirring for 2 h a homogeneous white solution was obtained (200 mL), sealed and sterilized (autoclave, with a filling fraction of 0.8 and kept at 120°C for 10 h). The white precipitate was formed, collected by filtration and washed with DI water and ethanol (in turn, three and two times respectively) to remove residues. The resultant Cu/ZnO nanostructure was dried in an oven at 80°C and then left on air for 12 h. 25 mg of GO powder was added to 50 mL deionized (DI) water under ultrasonic dispersion for 30 min. 0.4 g Cu/ ZnO (as synthesized) and 30 mL DI water were added to the GO solution and sonicated for 30 min. The mixture was transferred to a 100 mL Teflon liner and kept at 160°C for 12 h (sterilization). The white color of Cu/ZnO changed to dark gray. Meanwhile, GO were thermally reduced after the hydrothermal process, the gray precipitate was filtered and washed several times with DI water and ethanol separately. The final product (Cu/ZnO/rGO photocatalyst) (4 mol% of Cu and 6 mass% of rGO) was left in an oven at 70°C for 10 h. Pure ZnO nanostructures were synthesized using the same procedure without addition of copper nitrate hydrate as controls.

### Characterization of Cu/ZnO/rGO photocatalyst

The morphology of the nanostructured samples was characterized by field emission gun scanning electron microscopy (FESEM, JEOL, JSM-5610LV) and transmission electron microscopy (TEM, JEOL, JEM-200CX). The crystal structure of the as-synthesized Cu/ZnO/rGO was determined by X-ray diffractometer (XRD, Bruker, D8 ADVANCE) using high-intensity Cu Karadiation (k = 1.54051 Å) at an accelerating voltage of 40 kV and emission current of 30 mA. The spectrum was recorded from the region of 2θ (20–80°). UV-visible spectroscopy was used to evaluate the optical absorption in the range 300–850 nm (UV-Vis spectrophotometer, Evolution 220). X-ray photoelectron spectroscopy (XPS, ThermoFisher Scientific ESCALAB250Xi) was used to analyze the surface chemical state.

### Photocatalytic removal of aqueous ${\text{NH}}_{4}^{+}$-N

The photocatalytic removal of aqueous ammonia was performed in a cylindrical quartz batch photoreactor. A certain dose of photocatalyst was suspended in 100 mL NH_4_Cl aqueous solution. Prior to photocatalytic process, adsorption of ammonia in darkness for 30 min was performed to achieve adsorption equilibrium. The reaction solution was then irradiated under light illumination for 2 h under the xenon lamp (120 W, light with wavelengths 400–700 nm, visible light region). The high-pressure Hg lamp (125 W, with a peak light intensity at 254 nm, UV light region) was used to compare the ${\text{NH}}_{4}^{+}$-N removal efficiency of photocatalysts under different light sources. The distance between light source and photoreactor was set as 10 cm. The reaction solution was supplied with oxygen via bubbling (150 mL/min flow rate) to develop a consistent amount of dissolve oxygen. For comparison, a blank control without photocatalyst was also evaluated to quantify ${\text{NH}}_{4}^{+}$-N volatilization.

In this study, the effect of operational parameters including initial ammonia concentration, initial pH and Cu/ZnO/rGO dosage on the photocatalytic degradation efficiency were investigated. For the evaluation of the initial pH, the photocatalytic oxidation of aqueous ${\text{NH}}_{4}^{+}$-N at different pH (4, 7, 9, 10, and 11) was performed by adding 2 g photocatalyst to 100 mL aqueous ammonia solution (50 mg/L). The pH was adjusted to desired values (4, 7, 9, 10, and 11) by an addition HCl or KOH solutions. To evaluate the impact of catalyst excess, different amounts of Cu/ZnO/rGO (0.2, 0.5, 1, 2, and 3 g/L) were dispersed in 100 mL aqueous ammonia solution (50 mg/L), the solution pH was adjusted to 10. The effect of the initial ammonia concentration (10, 30, 50, 70, and 100 mg/L) on the photocatalytic degradation of ammonia was investigated at 2 g/L of the photocatalyst and initial pH 10. All trials were performed at room temperature (25°C) using constant magnetic stirring, the illumination time was up to 2 h. A volume of 1 mL of the treated solution was sampled every 30 min and then immediately centrifuged at 8,000 rpm for 10 min to remove photocatalyst-originated particles prior analysis. The concentrations of ${\text{NH}}_{4}^{+}$-N, ${\text{NO}}_{3}^{-}$-N, and ${\text{NO}}_{2}^{-}$-N in the supernatant were measured via flow injection analysis on a Skalar San++ System (Skalar Co., The Netherlands). For the catalyst stability test, leaching of Cu^2+^ and Zn^2+^ ions in the supernatant were analyzed by inductively coupled plasma mass spectroscopy (ICP-MS) (Thermo Scientific, Waltham, MA). To examine reusability of Cu/ZnO/rGO, the photocatalyst was recovered by centrifugation and washed with DI water prior re-application. The consecutive photocatalytic experiments were conducted for five runs. All trials were conducted in triplicate. And the data were expressed as the means with standard deviation (SD). The significance of the difference between the treatments means was assessed by Tukey's multiple range tests. Differences at *P* < 0.05 were considered statistically significant.

### Treatment of real domestic wastewater by Cu/ZnO/rGO

The real wastewater used in the present study was collected from a manhole shaft in the residential area of Jiangsu Academy of Agricultural Sciences. The general characteristics of the wastewater were as follows: ${\text{NH}}_{4}^{+}$-N: 43.5 ± 0.76 mg/L; total nitrogen (TN): 47.6 ± 0.95 mg/L; total phosphorus (TP): 5.44 ± 0.36 mg/L and chemical oxygen demand (COD): 68.5 ± 1.2 mg/L. It is suggested that nitrogen mainly exists as ${\text{NH}}_{4}^{+}$-N. Cu/ZnO/rGO nanocomposite (2 g·L^−1^) was added to 100 mL wastewater in the photoreactor. Its performance in the simultaneous removal of P, COD, and N (${\text{NH}}_{4}^{+}$-N and TN) was evaluated in this investigation. After visible light illumination for 2 h, aqueous samples were analyzed three times. COD was measured using a COD analyzer (DR1010 COD, HACH, China). N (${\text{NH}}_{4}^{+}$-N and TN) and P were analyzed on a Skalar San++ System (Skalar Co., The Netherlands).

## Results and discussion

### Characterization of photocatalysts

**Figure 1:**
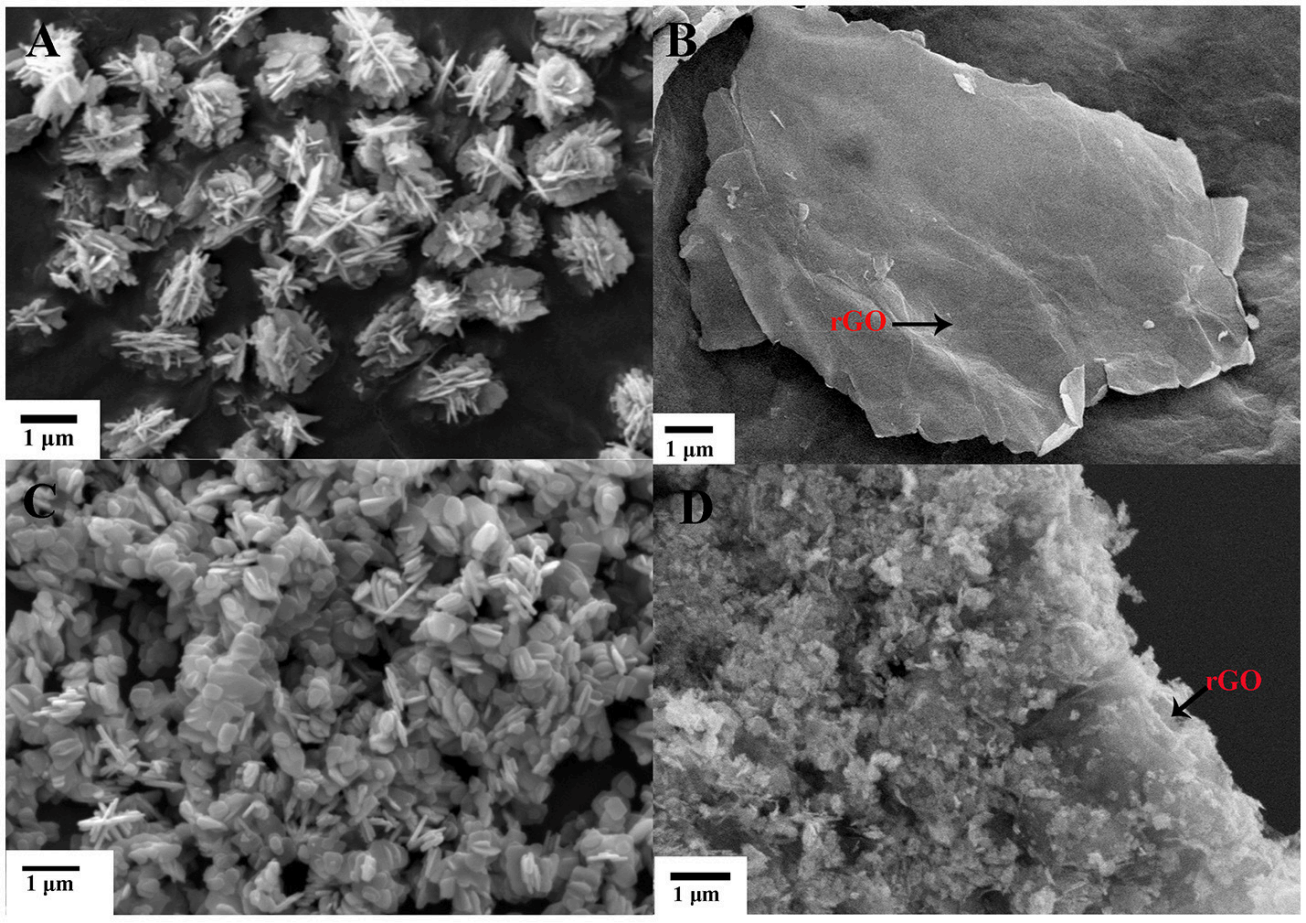
SEM images of **(A)** ZnO, **(B)** GO, **(C)** Cu/ZnO, and **(D)** Cu/ZnO/rGO nanocomposite.

**Figure 2:**
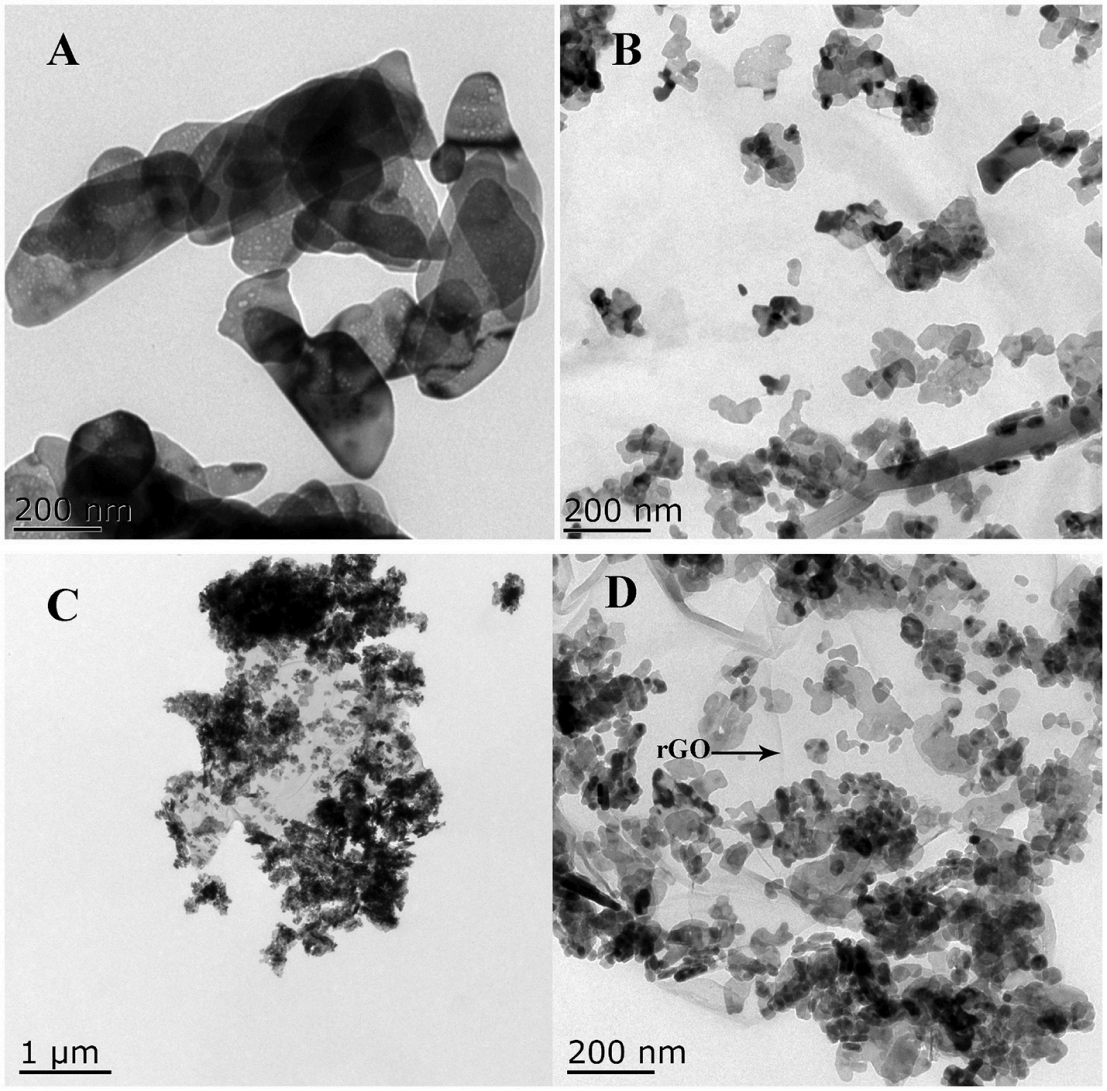
TEM images of **(A)** ZnO, **(B)** Cu/ZnO, **(C)** Cu/ZnO/rGO, and **(D)** enlarged image of Cu/ZnO/rGO.

Figures 1, 2 show the morphology of the as-synthesized pure ZnO, Cu/ZnO, and Cu/ZnO/rGO nanostructures (SEM, TEM). It is observed that pure ZnO exhibits nanosheet-like structure with a rough surface and irregular shape (Figures 1A, 2A). The images reveals that numerous ZnO nanosheets (about 50–100 nm) aggregated and assembled flower-like microsphere structures (with the size ranging from 500 nm to 1 μm). The morphology and size considerably changed by the addition of Cu^2+^. Different morphologies of these Cu/ZnO nanostructures including nanospheres, nanorods and nanosheets were observed (Figures 1C, 2B). Cu doping also reduced the agglomeration of ZnO. The average nanostructures size decreased (about 10–60 nm) when Cu^2+^ was incorporated to the ZnO matrix. These variations in morphology and size can be attributed to potential changes in the ZnO nucleation and growth process. The decrease in the average size of Cu/ZnO implies that the overall growth rate decreased in all dimensions for the treatment with Cu^2+^ doping. Since the ionic radii of Cu^2+^ ions is different from that of Zn^2+^, the presence of Cu^2+^ ions in the precursor solution induces a thermodynamic barrier that hinders the ZnO nucleation rate and impedes Zn species from adsorption for further growth. Therefore, the further nanostructure growth at all ZnO dimensions decreases. It has been reported that doping ZnO with Ce and Cu ions can form Zn–Ce–Cu–O in the crystal lattice and decrease the size of the nanoparticles (Mary et al., 2015). The smaller particles can provide with increased reactive sites promoting photocatalysis. Smaller particles have the tendency to interact with nanoparticles, resulting to irregular shapes (Mary et al., 2015).

With the incorporation of rGO, the close interfacial connection between ZnO and rGO became well-defined. SEM and TEM images showed the formation of Cu/ZnO/rGO nanocomposite ranging from sub-micrometers to several micrometers (Figures 1D, 2C,D). The well-defined Cu/ZnO nanostructure anchors on the surface of rGO sheets, illustrating the excellent adhesion between rGO and Cu/ZnO nanostructures. It is evident that rGO effectively inhibits ZnO agglomeration. This close contact between rGO and ZnO is beneficial to the effective photo-generated charge transfer, effectively inhibiting the e^−^/h^+^ pair recombination, which is favorable for photocatalytic reaction. It was also observed that the morphology and the size range of the anchored ZnO nanostructures are not affected by the addition of the hydrothermally produced rGO. rGO acts as an ideal support for semiconductors due to its unique two dimensional structure, high electron conductivity and mobility, high surface area, and excellent chemical stability. It is also reported that rGO has a series of oxygen functional moieties, including hydroxyl, and epoxy groups on its panels as well as carboxyl and carbonyl groups at its edges (Fu et al., 2011). The presence of the functional groups on the surface of rGO acts as anchoring sites for ZnO nanoparticles and prevents from nanopaticles agglomeration (Najafi et al., 2017).

**Figure 3:**
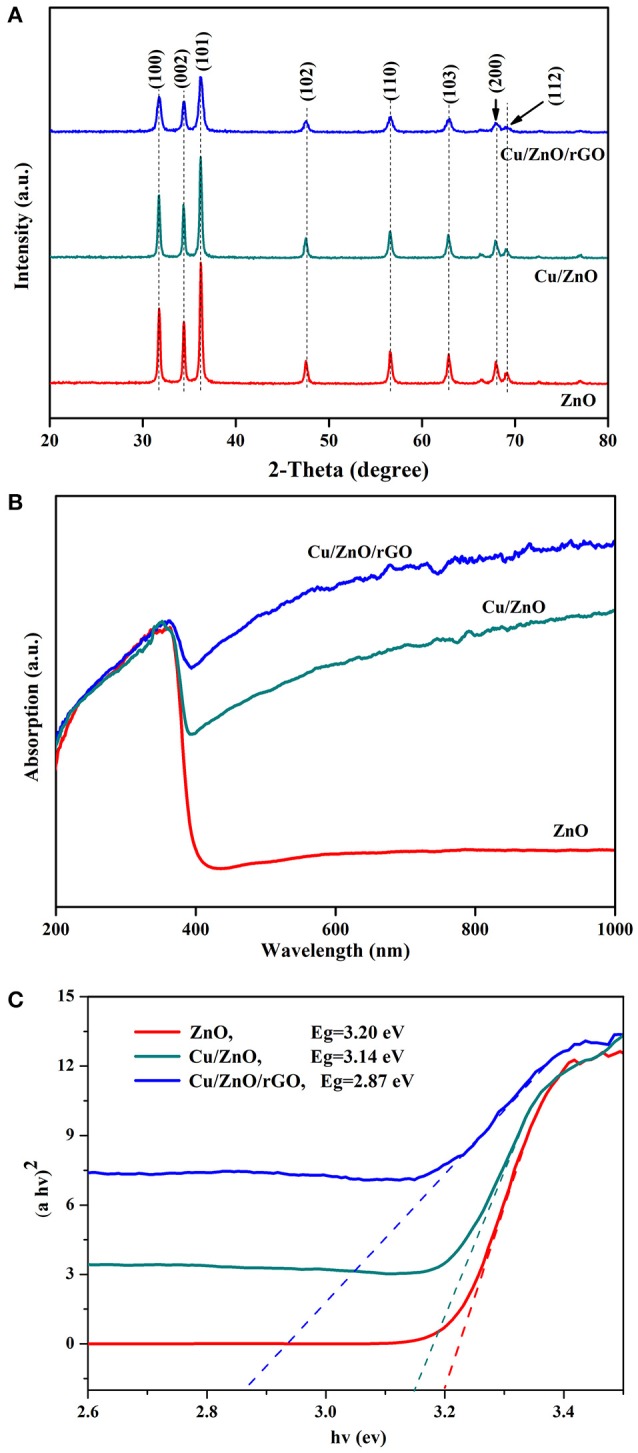
**(A)** XRD patterns of samples, **(B)** UV-Vis diffuse reflectance spectra of samples, and **(C)** Tauc's plots for ZnO, Cu/ZnO, and Cu/ZnO/rGO.

The crystal structural parameters of the synthesized ZnO, Cu/ZnO and Cu/ZnO/rGO nanostructures (Figure 3A) showed that all samples have almost identical patterns. The diffraction peaks exhibit a single phase hexagonal wurtzite structure without any impurities indicating Cu^2+^ and rGO do not change the crystalline structure of ZnO. The diffraction peaks (at 2θ = 31.8, 34.3, 36.1, 47.6, 56.6, 62.8, 68, and 69.1° Corresponding to the (100), (002), (101), (102), (110), (103), (200), and (112) planes) well match with the hexagonal wurtzite structure of crystalline ZnO (00-036-1415). The sharp and intense peaks in all samples indicate high single crystallinity. No additional peak from copper or its complex oxides was detected in the recorded patterns within the detection limits. This indicates that the doping does not alter the original structure by adding an additional phase. Decreased diffraction peak intensities were observed in Cu/ZnO and Cu/ZnO/rGO due to the substitution of Cu ion by ZnO crystal lattice at the Zn^2+^ sites. This replacement can be attributed to the fact that ionic radii (0.73 Å) of Cu^2+^ is close to that of Zn^2+^ (0.74 Å) (Meshram et al., 2016). It is difficult to see the rGO peaks in Cu/ZnO/rGO which may be due to the low content of rGO as well as the variation of the stacking mode of r caused by the incorporation of the ZnO nanostructures.

The internal changes of the synthesized nanocomposite samples are also reflected at their light absorption, based on UV–Vis diffuse reflectance spectra (Figure 3B). Pure ZnO shows a strong absorption peak at ≈360 nm (typical band-to-band transition characteristics of the wurtzite hexagonal ZnO) (Zhao et al., 2017). No apparent absorption was observed in the visible light region. It is known that ZnO consists of O *2p* orbital of valence band (VB) and Zn *4s* orbital of conduction band (CB). The distance between the VB (O 2p) and the CB (Zn 4s) of pure ZnO is ~3.2 eV, hence, it can absorb a major part of UV light (Xia et al., 2014; Lee et al., 2016). When Cu^2+^ ions are doped to the ZnO, an additional broad absorption peak in the visible light (range 500–800 nm) was observed due to a reduction in the band gap. The optical absorption measurement supports the Cu substitution in the ZnO lattice. As an impurity in the ZnO lattice, the localized *d*-electrons of Cu^2+^ ions narrow the ZnO VB and CB gap by forming strong Cu (*3d*)-O (*2p*) and Cu (*3d*)-Zn (4*s*) bonds (Xia et al., 2014). Furthermore, with the addition of rGO, the adsorption in the visible light region is enhanced compared to pure ZnO and Cu/ZnO nanostructures. rGO is a zero-gap semiconductor, the interaction of ZnO with rGO can generate an electronic energy level below the CB of ZnO (Bolotin et al., 2008). Thus, the Cu/ZnO/rGO nanocomposite requires decreased band gap energy and an increased light absorption. Moreover, the increased absorption in the visible light is also ascribed to the black body properties of rGO sheets. The white color of ZnO changed to dark gray after rGO introduction, indicating that the addition of rGO leads to an enhancement of background absorption in the visible light region (for Cu/ZnO/rGO nanocomposite). The band gaps of the as-prepared photocatalysts were calculated using the following formula for near-edge optical absorption of semiconductors:

(12) $$\begin{array}{r} {\left(\frac{\alpha hv}{A}\right)}^{2}=\left(hv-Eg\right) \end{array}$$

Where α is the absorption coefficient, *hv* is the photon energy, A is a constant. Figure 3C shows the plot of (α*h*ν)^2^ vs. *h*ν. The calculated *Eg* values are 3.20, 3.14, and 2.87 eV for ZnO, Cu/ZnO and Cu/ZnO/rGO, respectively. It can be clearly seen that Cu/ZnO/rGO composite has the lowest band gap value (2.87 eV), which is beneficial for the absorption of the visible light absorption.

**Figure 4:**
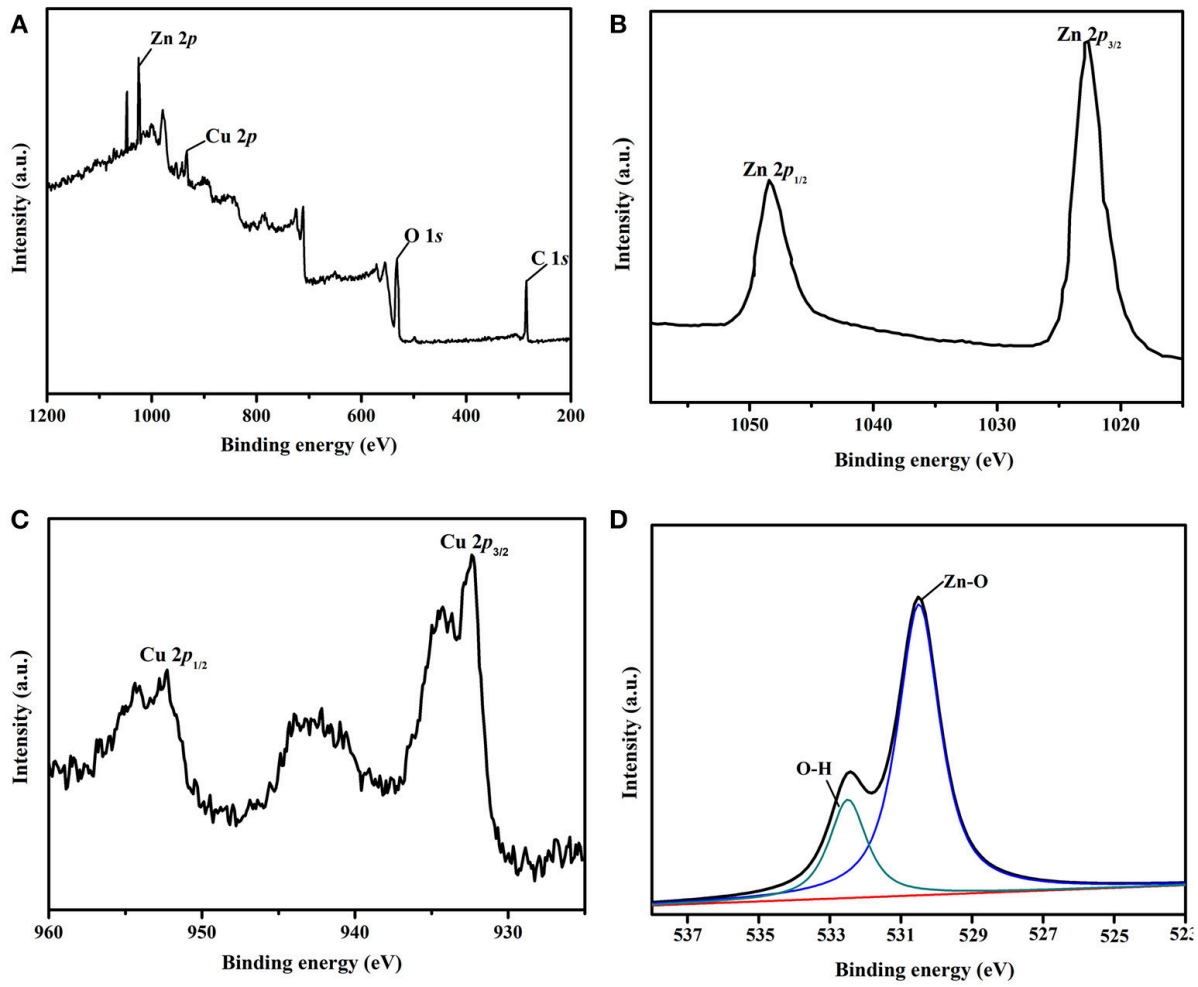
**(A)** XPS spectrum of Cu/ZnO/rGO, **(B)** high-resolution spectrum of the Zn 2p region, **(C)** high-resolution spectrum of the Cu 2p region, and **(D)** and high-resolution spectrum of the O 1s region.

The full scan XPS spectrum and high-resolution XPS spectra of Zn 2p, Cu 2p, and O 1s were carried out to clarify the elemental composition and chemical state of the as-synthesized Cu/ZnO/rGO nanocomposite. It is obvious from the Figure 4A that the detected peaks were assigned to Zn, Cu, O, and C. As also shown in Figure 4B, the intense character peaks centered at 1,022.62 eV (Zn 2p3/2) and 1,048.42 eV (Zn 2p1/2). This clearly indicates the oxidation state of Zn atoms. The Cu 2p binding energies of the as-prepared samples were 932.15 eV (Cu 2p3/2 and 952.36 eV (Cu 2p1/2) (Figure 4C), respectively, which also proved the oxidation state of Cu atoms in the Cu/ZnO/rGO. From O 1s spectra (Figure 4D), it was observed that the two peaks belonging to Zn-O, and O-H bonding located at 530.69 and 532.51 eV, respectively.

Combining the results from XRD spectroscopy, SEM, TEM images, UV-Vis, and XPS analyses, the Cu/ZnO/rGO nanocomposite possesses a smaller and better-dispersed ZnO on rGO with notably enhanced absorption in the visible light region. This suggests the theoretically, at least, improvement of the visible-light photocatalytic activity for aqueous ammonia removal in wastewater.

### Photocatalytic removal of ${\text{NH}}_{4}^{+}$-N with Cu/ZnO/rGO under UV and visible light

**Figure 5:**
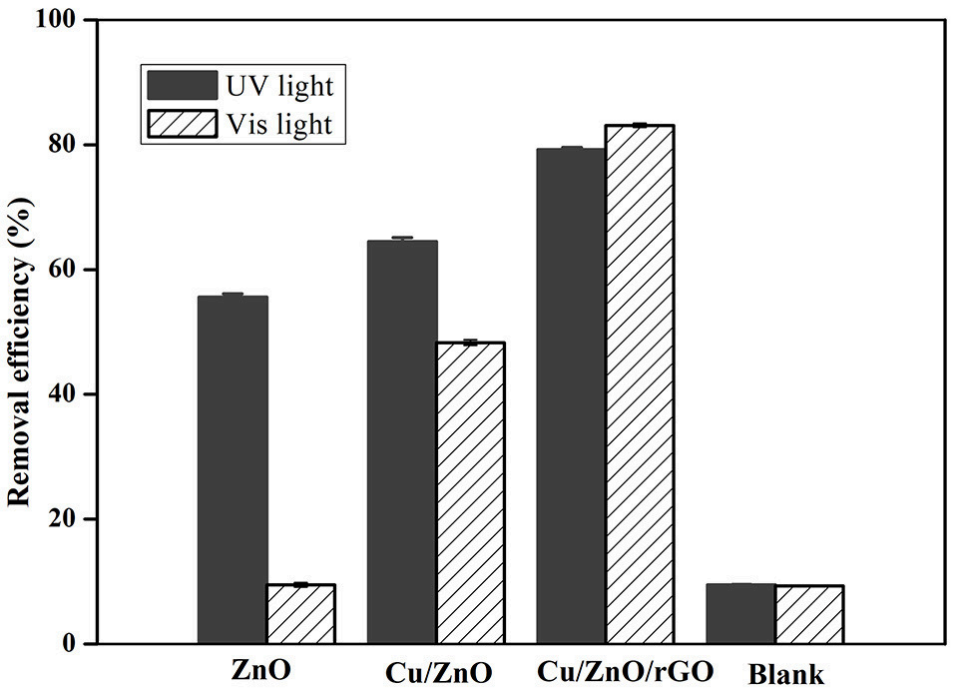
Photocatalytic removal efficiency of ${\text{NH}}_{4}^{+}$-N under UV and Vis irradiation with no catalyst (blank), ZnO, Cu/ZnO, and Cu/ZnO/rGO.

The aim of this study is to develop a new photocatalyst for aqueous ammonia degradation under visible light. The photocatalytic activities of the as synthesized photocatalysts were compared under a xenon (400 nm cutoff filter, visible light region) and Hg lamp (UV light region). Controls without photocatalysts showed that the concentration of aqueous ${\text{NH}}_{4}^{+}$-N reduces by 10% (Figure 5), indicating the volatilization is not the major contributor in the ${\text{NH}}_{4}^{+}$-N removal process. All the photocatalysts (pure ZnO, Cu/ZnO, and Cu/ZnO/rGO) exhibited good photocatalytic activities for NH_4_-N treatment in the UV light region. As shown in Figure 5, the removal efficiency under UV light with ZnO, Cu/ZnO, and Cu/ZnO/rGO were 55.6, 64.5, and 79.2% respectively. Pure ZnO nanoparticles showed negligible photocatalytic activity on ammonia under visible-light, which is due to the large band gap of ZnO and the lack of absorption in the visible region. It is also observed that the Cu doped ZnO nanocomposite exhibits higher photocatalytic activities than pure ZnO, especially in the visible light region. In the photocatalytic process, the photo-generated holes in the VB and the electrons from the CB could participate in the oxidation of aqueous ${\text{NH}}_{4}^{+}$-N (Yuzawa et al., 2012). From literature, dopant ions incorporated in the ZnO matrix can modify the energy band of ZnO by creating new electron energy levels within the bandgap (Xia et al., 2014; Sharma et al., 2017). The photo-generated electrons could easily transfer from the CB of ZnO to the localized dopant energy levels and get trapped by the dopant ions. In the meantime, the photo-generated holes were left in the VB of ZnO and migrated to the catalyst surfaces. Therefore, the recombination of the electron and the hole could be inhibited. The excited electrons and holes subsequently could produce more reactive oxygen species (ROSs, mainly •OH and •${\text{O}}_{2}^{-}$ radicals) to degrade ammonia, resulting in the improved photocatalytic activity under visible light. The decrease in the particles size will increase the specific surface area and provide with larger active surface, which consequently would lead to a higher interfacial charge carrier transfer for photocatalysis. Cu ions doped with ZnO or TiO_2_ have also been reported capable to enhance the methyl orange, phenol and humic substances degradation efficiency (Maleki et al., 2015; Zhang et al., 2015; Meshram et al., 2016).

The ${\text{NH}}_{4}^{+}$-N removal efficiency was substantially enhanced with rGO supporting. It is obvious that Cu/ZnO/rGO composite catalysts exhibited the highest photocatalytic degradation efficiency, with removal efficiency of 79.2% in UV light and 83.1% under visible light irradiation. The influence of rGO at the photocatalytic ammonia removal enhancement can be explained as follows: (i) In the Cu/ZnO/rGO composite, ZnO is one of the most efficient electron donors while rGO is a relatively effective electron acceptor. Irradiated with visible photon energy, photo-excited electrons and holes from ZnO were extracted by the rGO bridges through interfacial interactions. The high conductivity of rGO sheets enables quick charge transfer prolonging the charge carriers life-time, suppressing the h^+^/e^−^ recombination (Zhao et al., 2017). These interactions can effectively enhance the generation of active radical species under photo irradiation which are eventually responsible for the photocatalytic decomposition of ammonia. (ii) From the obtained results, rGO nanosheets have the ability for ammonia adsorption due to their larger specific surface areas and functional oxygen groups. rGO could also prevent ZnO agglomeration enhancing the adsorption of ammonia molecules. The ammonia molecules adsorbed on photocatalyst are more favorable to interact with the ROSs than those in the solution form. (iii) Moreover, the high optical transparency of rGO facilitates light harvesting and subsequently enhances the power conversion efficiency. Furthermore, increase of the rGO concentration over 6% mass adversely affects the ${\text{NH}}_{4}^{+}$-N removal efficiency (Figure S1). The reduced efficiency is most likely caused by rGO overloading. The excessive rGO could act as a block restricting the light absorption of ZnO and encouraging the electron hole recombination. Similar behavior on the effect of rGO dosage has been reported previously, where rGO overloading caused a negative influence over the catalytic performance (Ranjith et al., 2017).

**Figure 6:**
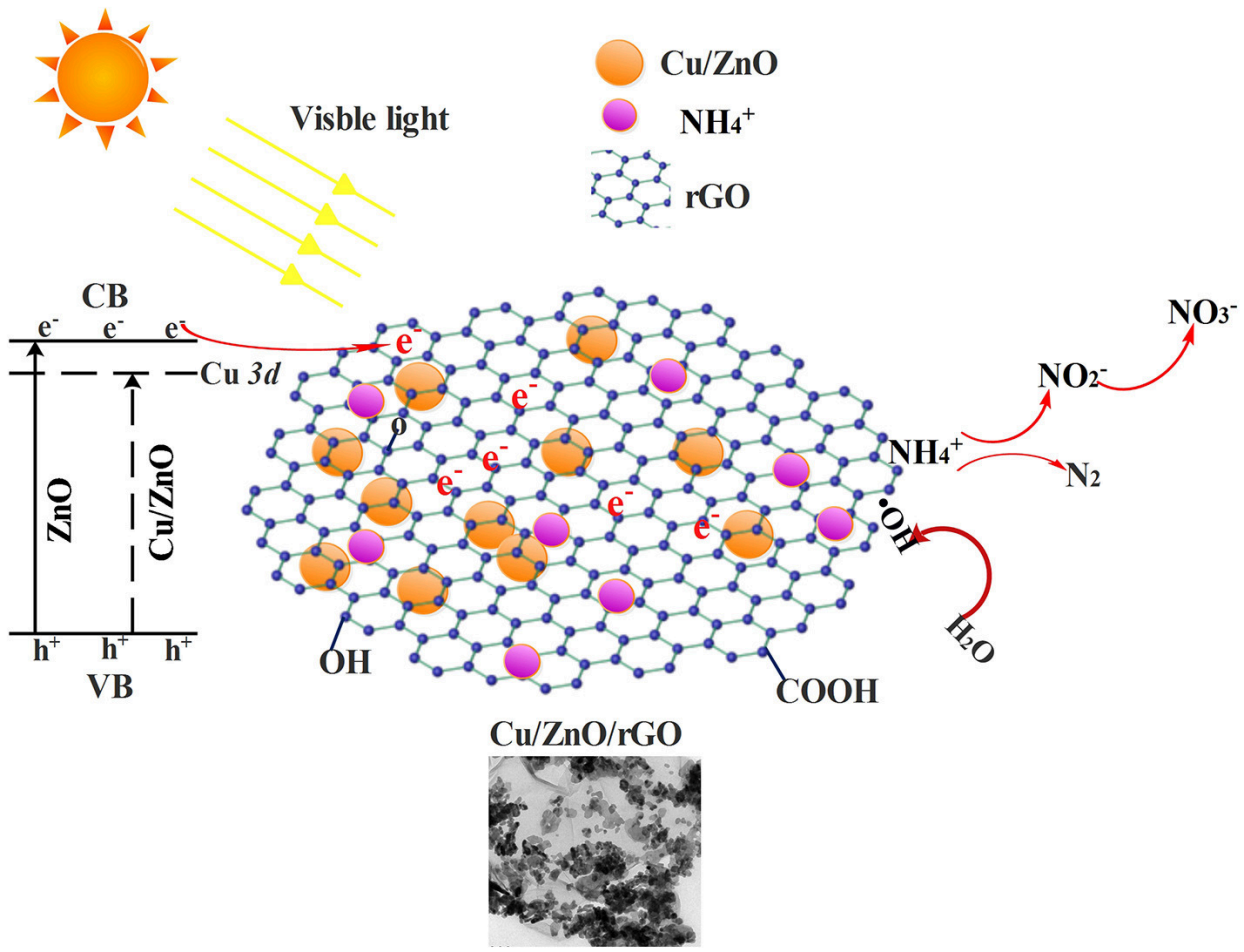
Potential reaction mechanism for ${\text{NH}}_{4}^{+}$-N photo degradation via Cu/ZnO/rGO.

In conclusion, combined with Cu doping and rGO supporting, photocatalytic activity of ZnO under visible-light irradiation is enhanced. A possible reaction mechanism and the photodegradation process are illustrated in Figure 6.

**Figure 7:**
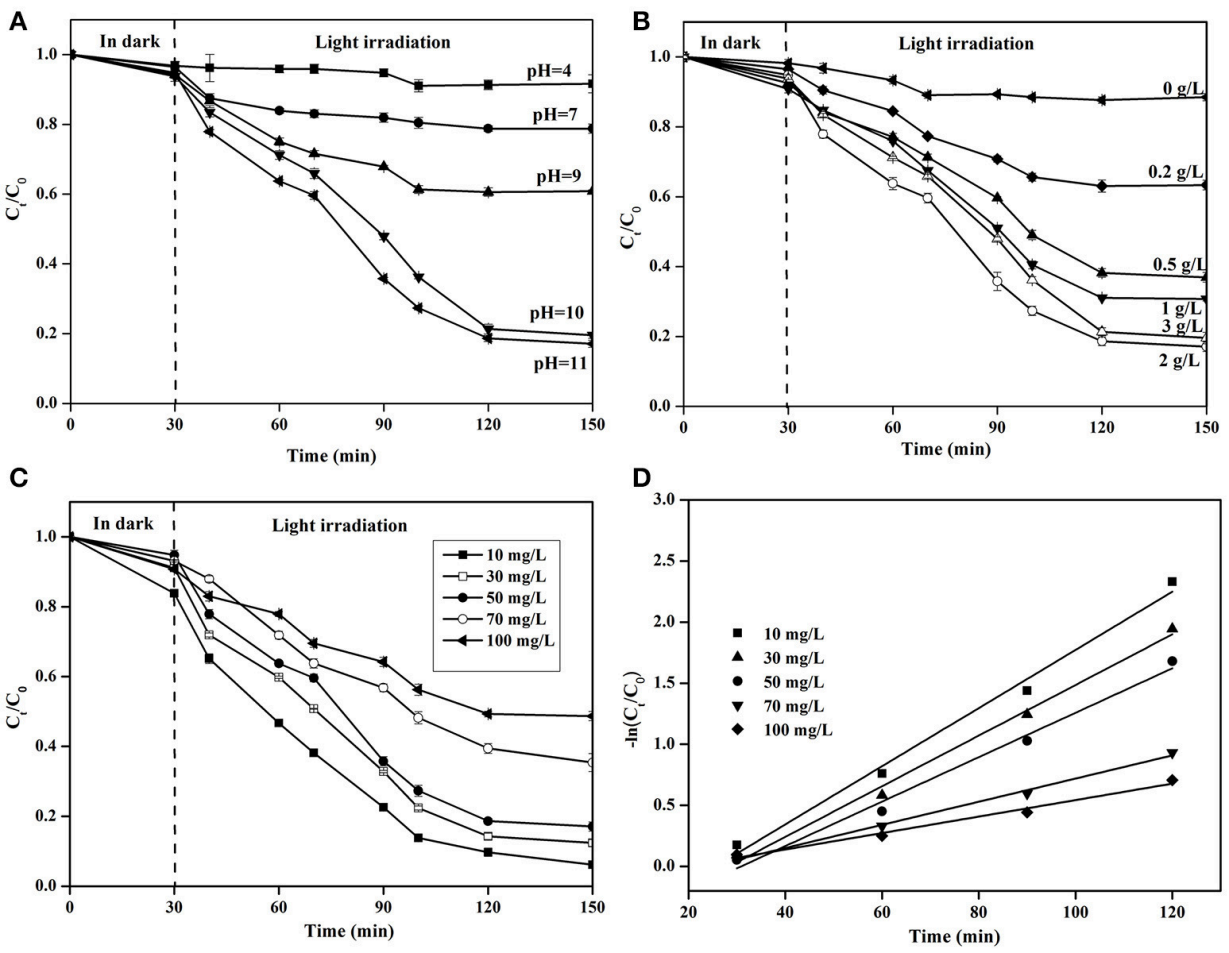
Photocatalytic ${\text{NH}}_{4}^{+}$-N removal efficiency **(A)** at different pH, **(B)** at various Cu/ZnO/rGO dosages, and **(C)** at various ${\text{NH}}_{4}^{+}$-N concentrations, **(D)** overall photocatalysis reaction kinetics.

pH is an vital parameter in photocatalytic reactions (Shibuya et al., 2015). For the investigation of the photocatalytic ${\text{NH}}_{4}^{+}$-N removal with Cu/ZnO/rGO at various pH (4.0 to11.0), the catalyst and the ammonia solution were kept in dark for 30 min then excited under light illumination for 2 h. The decrease of ${\text{NH}}_{4}^{+}$-N in the dark was less than 10%, and the reduction related to the physicochemical adsorption property of the photocalysts. From Figure 7A, Cu/ZnO/rGO demonstrated higher ${\text{NH}}_{4}^{+}$-N removal efficiency in alkaline conditions rather than in neutral or acidic ones. The concentration of ${\text{NH}}_{4}^{+}$-N remained unchanged at pH 4. The ${\text{NH}}_{4}^{+}$-N removal efficiency increased as per pH rising and reached the maximum at a pH >10. This is explained by the fact that pH influences not only the existing forms of the ${\text{NH}}_{4}^{+}$-N in solution but also the photocatalyst's surface charge properties (Shibuya et al., 2015). Firstly, ionized ammonium (${\text{NH}}_{4}^{+}$) and un-ionized ammonia (NH_3_) are the main types of ammonia nitrogen (at equilibrium in water with a pKa of 9.26). It has been reported that ${\text{NH}}_{4}^{+}$ could not be oxidized by the VB holes (${\text{h}}_{\text{VB}}^{+}$) or hydroxyl radicals (·OH). (Lee et al., 2002). When the solution pH is higher than 9.26, the dominant species of aqueous ammonia is NH_3_. The proportion of ${\text{NH}}_{4}^{+}$ is reversely proportional with the solution pH when < 9.26 (Sun et al., 2015). Thus, ammonia degradation efficiency decreases in neutral or acidic solutions. When the solution pH dropped to 7, almost all aqueous ammonia was in the form of ${\text{NH}}_{4}^{+}$, and impossible to be photocatalytically oxidized. Secondly, the number of hydroxide groups (OH^−^) increases with the pH increase, and more hydroxyl radical (·OH) can be generated by Cu/ZnO/rGO, resulting in the promotion of the ${\text{NH}}_{4}^{+}$-N removal efficiency. Thirdly, the surface of Cu/ZnO/rGO catalysts is maintained positively charged in acidic conditions, a condition unfavorable for the adsorption of ammonium molecules at the catalyst surface. Moreover, the space steric hindrance of NH_3_ is smaller than the one of ${\text{NH}}_{4}^{+}$, which is more conducive to the reaction of NH_3_ with ·OH (Luo et al., 2015). This finding is aligned with the theoretical relationship between pH and ammonia photocatalysis (Wang et al., 2014; Shibuya et al., 2015).

The optimum amount of catalysts is essential for effective photocatalytic removal of ammonia. Figure 7B shows the effect of Cu/ZnO/rGO dosage on photocatalytic performance under visible light. Increasing catalyst dosage from 0.2 to 2 g/L, ammonia degradation increases from 31.5 to 83.2% respectively. When the photocatalyst dosage is kept low, the production of ROSs is limited. Dosage increase could offer with more accessible active sites to be exposed to light irradiation and generate more ROSs leading to the enhanced photocatalytic activity. Further increase in the amount of Cu/ZnO/rGO (3 g/L) leads to a removal efficiency reduction. This is attributed to the excessive amount of photocatalysts that could reduce light transmittance and decrease the light penetration. Moreover, a large amount of catalysts could form larger agglomerates and consequently decrease the active catalyst surface. Therefore, a Cu/ZnO/rGO overdose limits photocatalytic performance. The optimal concentration of Cu/ZnO/rGO in the trial was found 2 g/L.

The initial ammonium concentration has a profound impact on the removal rate. As shown on Figure 7C, at high initial ${\text{NH}}_{4}^{+}$-N concentration a significant decrease (*P* < 0.05) in the rate of ammonium degradation was observed. This is due to the relationship of the rate of ammonia photo-degradation to the availability of catalytic sites on the catalyst surface that dictates the formation of ·OH. At this study, the amount of Cu/ZnO/rGO was kept constant, thus the high initial ammonia concentration could occupy a greater number of Cu/ZnO/rGO active sites available for degradation in the presence of excessive “influent” concentration. Thus, the formation of ·OH on the catalyst surface was inhibited. This results in decreased removal rates and subsequently to longer retention times to complete degradation. Figure 7C indicates that the percentage of degradation varies from 89.2 to 45.0% for 10–100 mg/L respectively. The kinetic studies of the photocatalytic degradation of ammonia using Cu/ZnO/rGO at different initial concentrations were also investigated using the pseudo-first-order kinetic reaction:

(13) $$\begin{array}{r} \ln\left\{\frac{C_{0}}{C}\right\}=Kt \end{array}$$

Where *K* (min^−1^) is the first-order rate constant, which is calculated from the slope of ln (*C*_0_*/C*) vs. *t* plots, *t* is the irradiation time, *C*_0_ is the initial ${\text{NH}}_{4}^{+}$-N concentration at the beginning of the photocatalytic reaction, C is the ${\text{NH}}_{4}^{+}$-N concentration at different irradiation time. A linear relationship between the ln (*C*_0_*/C*) and *t* (Figure 7D) shows that the rate constant values *K* (min^−1^) decreases with increased ammonia concentration. The values of rate constants for 10, 30, 50, 70, and 100 mg/L of ${\text{NH}}_{4}^{+}$-N are estimated to be 0.0238 min^−1^, 0.02073 min^−1^, 0.01819 min^−1^, 0.00947 min^−1^, and 0.00647 min^−1^, respectively.

**Figure 8:**
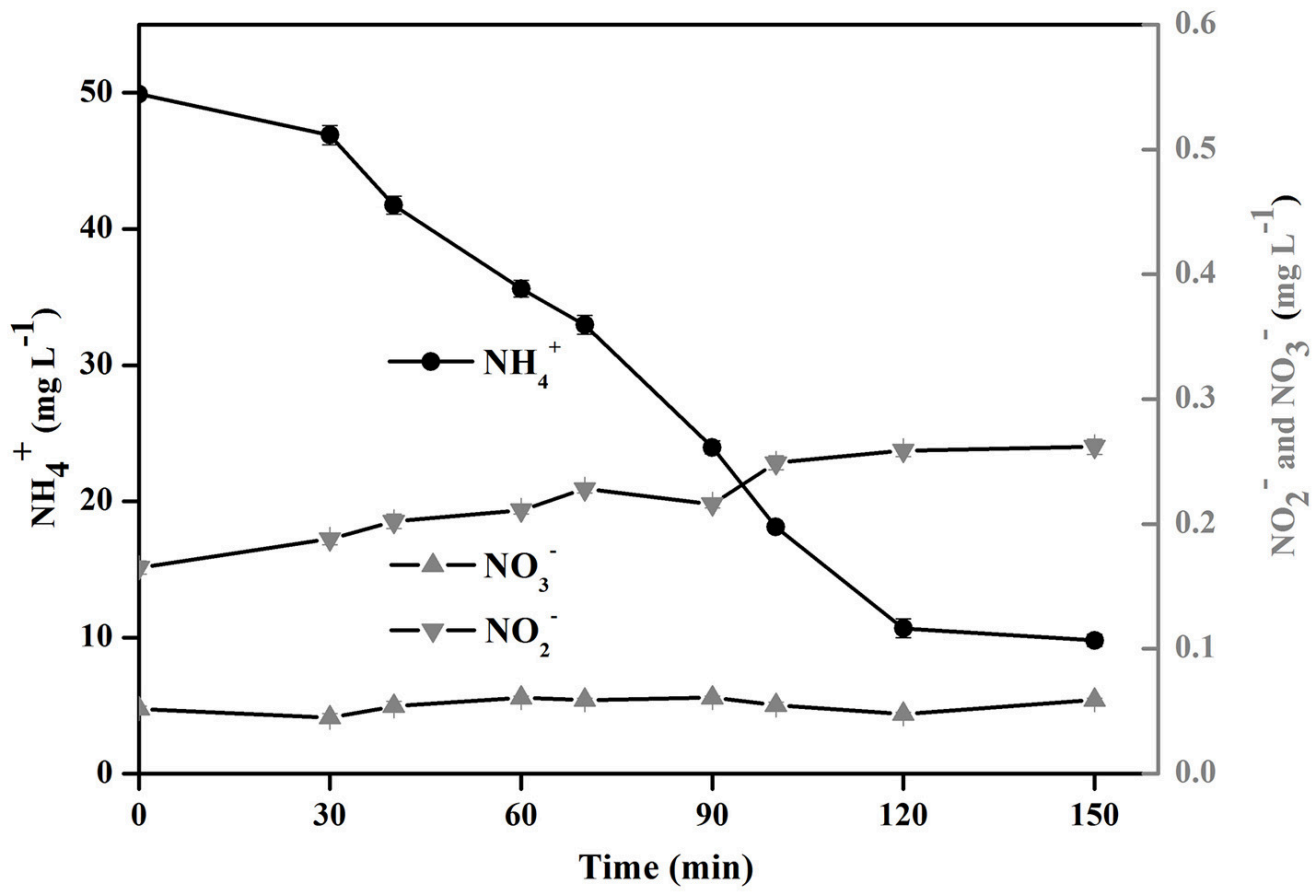
Fate of ${\text{NH}}_{4}^{+}$-N, ${\text{NO}}_{2}^{-}$-N, and ${\text{NO}}_{3}^{-}$-N using Cu/ZnO/rGO for the photocatalytic oxidation of ammonia.

As shown in Reaction Schemes I and II, nitrogen gas (N_2_), nitrite (${\text{NO}}_{2}^{-}$-N), and nitrate (${\text{NO}}_{3}^{-}$-N) are the plausible by-products of the photocatalytic removal of aqueous ${\text{NH}}_{4}^{+}$-N. To further confirm the mechanism of the ${\text{NH}}_{4}^{+}$-N oxidation with Cu/ZnO/rGO the fate of the inorganic N atoms (including ${\text{NH}}_{4}^{+}$-N, ${\text{NO}}_{2}^{-}$-N, and ${\text{NO}}_{3}^{-}$-N) during the process were quantified. Figure 8 shows that concentration of aqueous ${\text{NH}}_{4}^{+}$-N decreased from 50 mg/L to 8.5 mg/L after 2 h treatment. During the degradation process, only minor amounts of ${\text{NO}}_{3}^{-}$-N (0.43 mg/L) and ${\text{NO}}_{2}^{-}$-N (0.262 mg/L) were detected. Considering the weak volatilization of aqueous ${\text{NH}}_{4}^{+}$-N (about 4.8 mg/ L, control experiment without catalyst) it is proposed that ~70% of ${\text{NH}}_{4}^{+}$-N is converted to N_2_ according to the N-mass balance. The formation of nontoxic N_2_ is an advantage of the specific process.

**Figure 9:**
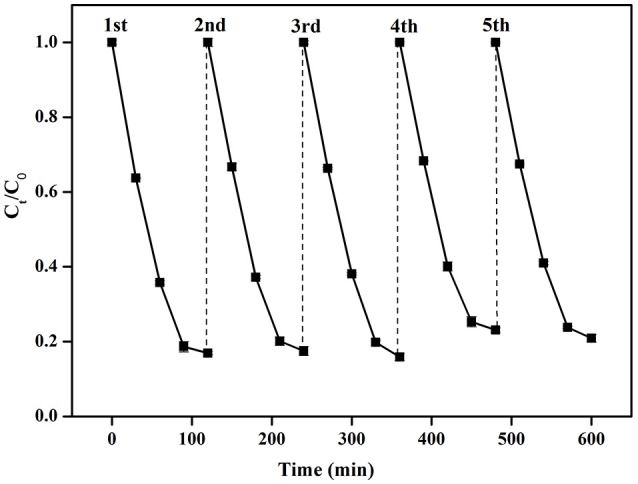
Repeated photocatalytic oxidation of ${\text{NH}}_{4}^{+}$-N by Cu/ZnO/rGO.

The stability of a photocatalyst is a crucial parameter for the evaluation of an agent as per its practical application. The stability of Cu/ZnO/rGO was evaluated by a successive cyclic experiment performed using 200 mg Cu/ZnO/rGO in 100 mL ammonia solution with an initial concentration of approximately 50 mg·L^−1^ under visible light irradiation. Figure 9 shows that the photocatalyst did not exhibit any obvious loss of activity, and high ${\text{NH}}_{4}^{+}$-N efficiency of Cu/ZnO/rGO was maintained even after five successive trials. Specifically, the photocatalytic was reduced by up to ~0.6%. Therefore, Cu/ZnO/rGO nanocomposite has a stable photocatalytic activity with a relatively satisfactory recyclability and reusability. The release of the Zn^2+^ and Cu^2+^ ions from the Cu/ZnO/rGO photocatalysts was also measured via ICP. The results pointed that Zn^2+^ and Cu^2+^ ions were not detected at any of the five-cycles. Moreover, the XRD analysis revealed that the structure of Cu/ZnO/rGO remained the same before and after the photocatalysis (Figure S2). The above indicate the good degree of stability of the Cu/ZnO/rGO underlying its high potential in practical applications.

In addition, we also compare our ${\text{NH}}_{4}^{+}$-N removal efficiency with some of the related literatures, it is shown that Cu/ZnO/rGO has similar ${\text{NH}}_{4}^{+}$-N removal efficiency with that of the reported catalysts (Table 1). The advantage of our investigation is that the removal efficiency was achieved within 2 h retention time. The short retention time dictates the size of the tank in water and wastewater treatment engineering. Subsequently the effective catalysts can reduce the capital cost of these applications paving the path toward sustainable treatment.

**Table 1:** Comparison of ammonia removal efficiency (%) using various photocatalysts.

**Photocatalysts**	**Light**	**Removal efficiency (%)**	**Main products**	**Time(h)**	**Ref**.
TiO_2_/perlite	UV	68	N_2_	3	Shavisi et al., 2014
Pd/N/ TiO_2_	Vis	80	N_2_	2	Sun et al., 2015
La/Fe/ TiO_2_	UV	64.6	${\text{NO}}_{3}^{-}$, ${\text{NO}}_{2}^{-}$, N_2_	5	Luo et al., 2015
ZnFe_2_O_4_/rGO	Vis	92.3	N_2_	4	Liu et al., 2017
SL g-C_3_N_4_	UV	80	${\text{NO}}_{3}^{-}$	6	Wang et al., 2014
TiO_2_-ZnO/LECA	Vis	95.2	/	3	Mohammadi et al., 2016
Cu/ZnO/rGO	Vis	83.2	N_2_	2	This work

### The COD, N, and P changes of actual domestic wastewater

**Figure 10:**
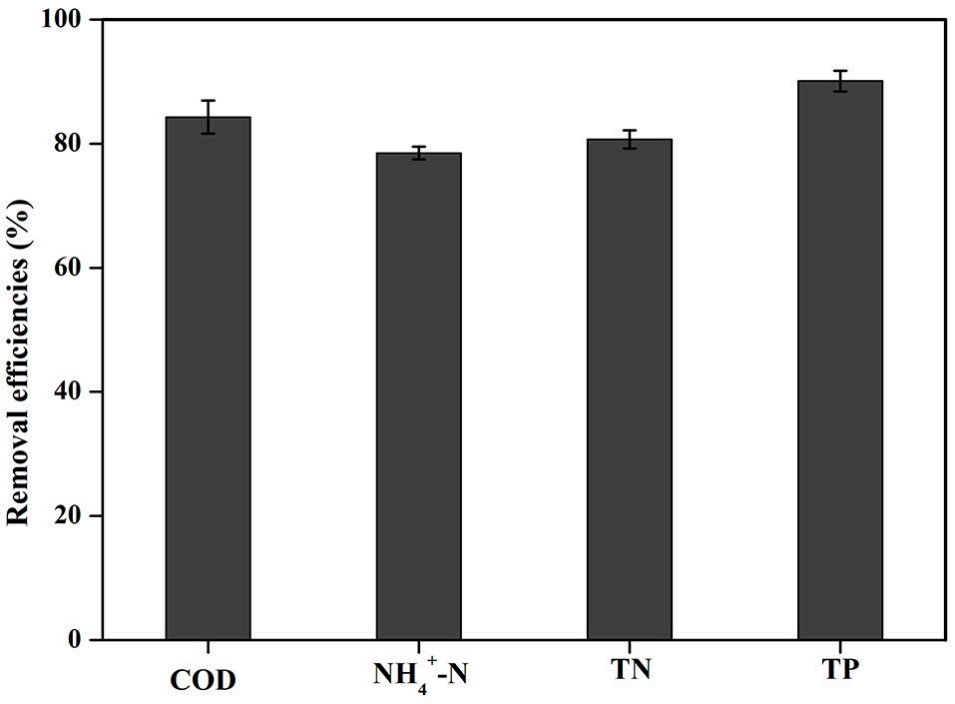
Treatment of real domestic wastewater by Cu/ZnO/rGO (COD, ${\text{NH}}_{4}^{+}$-N, TN, and TP removal efficiencies).

The applicability of Cu/ZnO/rGO nanocomposite in real domestic wastewater was also investigated. Our study clearly shows that Cu/ZnO/rGO exhibit highly efficient simultaneous removal of COD, N, and P (Figure 10). After treating wastewater with Cu/ZnO/rGO, the concentrations of COD were reduced by 57.7 mg/L (removal efficiency: 84.3%) by photocatalytic degradation. The removal efficiency of ${\text{NH}}_{4}^{+}$-N and TN were 78.5 and 80.7%, respectively. In addition, the concentration of TP decreased from 5.44 to 0.53 mg/L (removal efficiency: 90.3%). Based on these findings, it is clear that Cu/ZnO/rGO nanocomposite is an ideal agent for real wastewater treatment.

## Conclusions

In summary, Cu-doped ZnO/rGO nanocomposite was sufficiently prepared via a two-step hydrothermal method. The incorporation of Cu ions and rGO with ZnO broadened the response of the photocatalyst to visible light. The fabricated Cu/ZnO/rGO showed higher ammonia removal efficiency (>80%) than the pure ZnO under visible-light irradiation, and the major oxidation product was N_2._ The facile modification of ZnO by Cu ions and rGO can improve the ROSs production efficiency and further activate the interfacial catalytic sites, which account for the higher degradation efficiency. This study demonstrates that Cu/ZnO/rGO composite is a promising alternative for ${\text{NH}}_{4}^{+}$-N removal and real domestic wastewater treatment with effective application of solar energy. Further work is now underway to combine the Cu/ZnO/rGO nanocomposite with a suitable reactor to degrade pollutants for the water treatment in practice.

## Author contributions

SH, LX, and LY designed and conducted the experiments. PH, YF, and YY analyzed the data. SH, EP and LY wrote the paper.

### Conflict of interest statement

The authors declare that the research was conducted in the absence of any commercial or financial relationships that could be construed as a potential conflict of interest.
