# Supplementary material for: High Efficient Visible-Light Photocatalytic Performance of Cu/ZnO/rGO Nanocomposite for Decomposing of Aqueous Ammonia and Treatment of Domestic Wastewater
Source: Front Chem. 2018 Jun 12;6:219. doi: 10.3389/fchem.2018.00219 (PMC6005899; doi:10.3389/fchem.2018.00219)
Supplement: Supplementary file 1 [file Presentation_1.PDF]

Supplementary Material

High Efficient Visible-light Photocatalytic Performance of Cu/ZnO/rGO Nanocomposite for Decomposing of Aqueous Ammonia and Treatment of Domestic Wastewater

Shiying He[1], Pengfu Hou[1], Evangelos Petropoulos[2], Yanfang Feng[1], Yingliang Yu[1], Lihong Xue[1] and Linzhang Yang[1,*]

*** Correspondence:** Corresponding Author: E-mail: lzyang@issas.ac.cn

Supplementary Figure

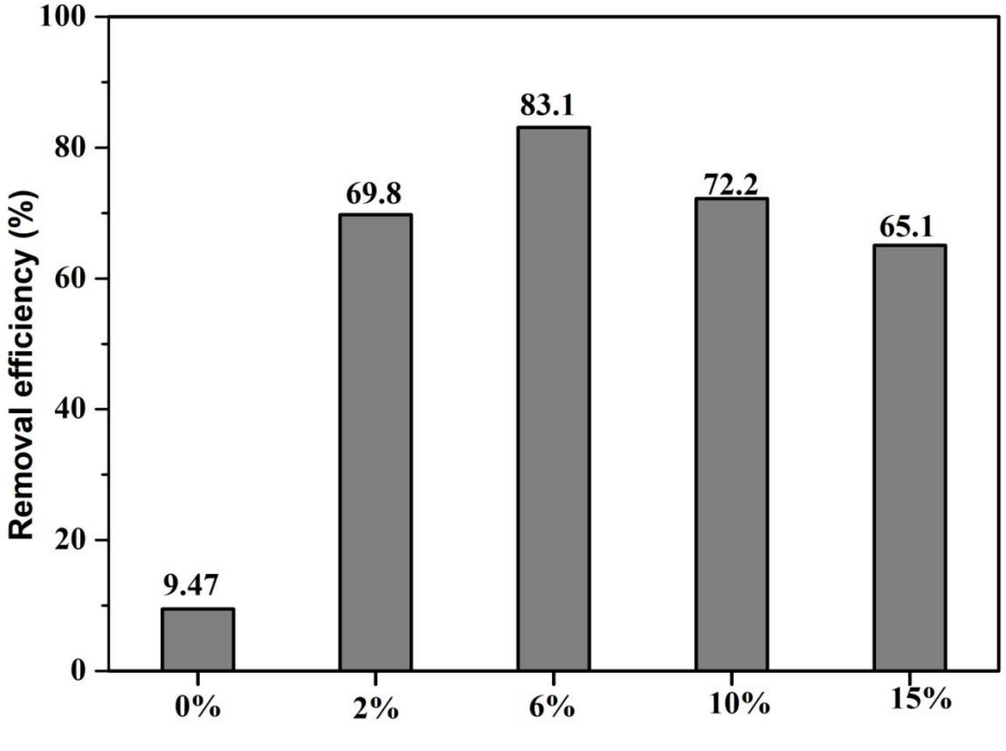

Figure S1. The effect of rGO concentration on photocatalytic performance of Cu/ZnO/rGO nanocomposite

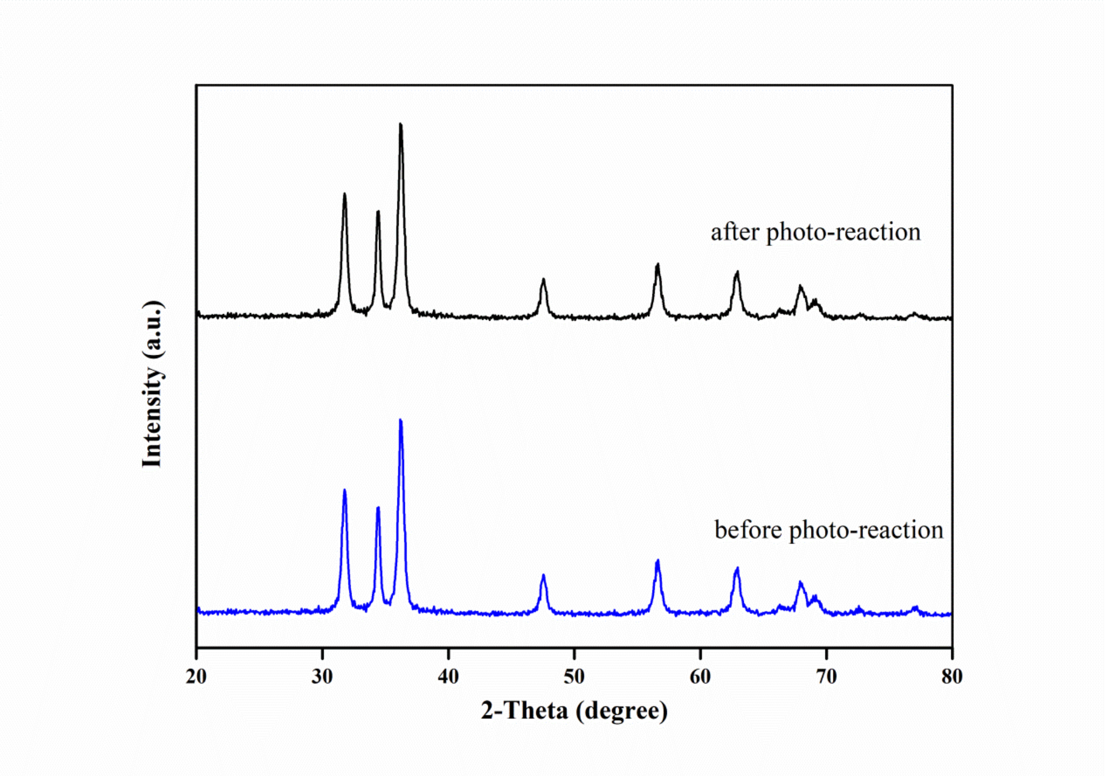

Figure S2. XRD analysis of Cu/ZnO/rGO before and after the photocatalysis
